# Commentary: The formal approach to quantitative causal inference in epidemiology: misguided or misrepresented?

**DOI:** 10.1093/ije/dyw227

**Published:** 2017-01-27

**Authors:** Rhian M Daniel, Bianca L De Stavola, Stijn Vansteelandt

**Affiliations:** 1 LSHTM Centre for Statistical Methodology and Medical Statistics Department, London School of Hygiene and Tropical Medicine, London, UK; 2 Department of Applied Mathematics, Computer Sciences and Statistics, Ghent University, Ghent, Belgium

Two recent articles, one by Vandenbroucke, Broadbent and Pearce (henceforth VBP)^1^ and the other by Krieger and Davey Smith (henceforth KDS),^2^ criticize what these two sets of authors characterize as the mainstream of the modern ‘causal inference’ school in epidemiology. The criticisms made by these authors are severe; VBP label the field both ‘wrong in theory’ and ‘wrong in practice’, and KDS—at least in some settings—feel that the field not only ‘bark[s] up the wrong tree’ but ‘miss[es] the forest entirely’. More specifically, the school of thought, and the concepts and methods within it, are painted as being applicable only to a very narrow range of investigations, to the exclusion of most of the important questions and study designs in modern epidemiology, such as the effects of genetic variants, the study of ethnic and gender disparities and the use of study designs that do not closely mirror randomized controlled trials (RCTs). Furthermore, the concepts and methods are painted as being potentially highly misleading even within this narrow range in which they are deemed applicable. We believe that most of VBP’s and KDS’s criticisms stem from a series of misconceptions about the approach they criticize. In this response, therefore, we aim first to paint a more accurate picture of the formal causal inference approach, and then to outline the key misconceptions underlying VBP’s and KDS’s critiques. KDS in particular criticize directed acyclic graphs (DAGs), using three examples to do so. Their discussion highlights further misconceptions concerning the role of DAGs in causal inference, and so we devote the third section of the paper to addressing these. In our Discussion we present further objections we have to the arguments in the two papers, before concluding that the clarity gained from adopting a rigorous framework is an asset, not an obstacle, to answering more reliably a very wide range of causal questions using data from observational studies of many different designs.

## An introduction to the formal approach to quantitative causal inference in epidemiology

### Labels

VBP characterize the mainstream view within what they call the ‘causal inference movement in epidemiology’ as belonging to the ‘restricted potential outcomes approach’, which they define to be the approach in which only the effects of exposures that correspond to currently humanly feasible interventions can be studied. KDS focus instead on DAGs (rather than potential outcomes) as the main target of their criticism. However, in many places they appear to (wrongly) conflate DAGs and potential ouctomes, and they certainly share the misconception that only currently humanly feasible interventions can be studied within this approach.

As we discuss later (see misconception 1), we strongly disagree with this characterization. We also don’t much like the term ‘movement’, and so—for want of a better label, and to avoid cumbersome repetitive descriptions—we’ll call the school of thought that both VBP and KDS have in their sight the ‘Formal Approach to quantitative Causal inference in Epidemiology’, or FACE. In the next sections we describe what we see as the core principles of this approach, with examples of where these have been illuminating and enabled causal analyses under less restrictive assumptions.

### The core principles of the FACE

The broad features that characterize the majority of the work done by the FACE are, having first thought carefully about the nature of the causal question to be addressed, to convert this into a precise quantity to be estimated (i.e. a causal estimand), typically using the notation of potential outcomes. The causal question one ideally wishes to address may often be replaced by a similar causal question that can more feasibly be addressed given the constraints of the data at hand. There is a trade-off here. No one wants ‘the right answer to entirely the wrong question’; indeed, this is what has led the FACE to recommend against ‘retreating into the associational haven’ but rather ‘to take the causal bull by the horns’.^3^ But presumably equally uncontroversial is the observation that ‘an entirely wrong answer to the right question’ is also futile. Arriving at a good compromise between these two competing concerns is one of the many important tasks facing applied researchers. Explicitly formulating the causal estimand may seem like an obvious first step, but one that is often ignored in applied practice where researchers may jump to modelling associations and presenting their results in terms of, for example, odds ratios or hazard ratios, while foregoing the more interesting and concrete scientific questions such as ‘what would the risk of this outcome be if one could eliminate the exposure?’ This clarity moreover allows one to be rigorous about the assumptions (e.g. consistency, conditional exchangeability and positivity) under which the estimand can be identified from the data at hand, and then for flexible estimation strategies to be developed that are valid under these assumptions. Finally, tools are recommended to assess quantitatively the sensitivity of the results to plausible departures from the assumptions, to aid interpretation, and to discuss possible misinterpretation, of the results. In the Supplementary material (available at *IJE* online) we give examples of causal estimands and describe the most commonly invoked assumptions for their identification in the context of a simplified investigation of the effect of maternal urinary tract infections during pregnancy on low birthweight.

### The advantages of adopting this approach

In many settings (problems involving time-dependent confounding and mediation are good examples^4–9^), the increased formality characteristic of the FACE has highlighted the implausibility of the assumptions (e.g. no ‘feedback’ between exposure and confounder) required for standard analysis strategies to give meaningful answers to the causal questions being posed, and has led to improved alternatives (e.g. g-methods) that are increasingly widely used in practice.^10–13^ The FACE has moreover given rise to an array of methods for nonlinear instrumental variable analysis^14–16^ and for nonlinear mediation analysis^9^^,^^17–23^ where only *ad hoc* and biased approaches existed before. Other examples where this approach has led to new insights and/or methods include the low birthweight and obesity ‘paradoxes’^24–27^ (see further discussion in ‘Example 2: Birthweight paradox’, below), the comparison of dynamic regimes,^28^ the impact of measurement error,^29^^,^^30^ noncompliance in clinical trials,^31^ distinguishing confounding from non-collapsibility^32^ and many more.

More recently, and looking to the future, the advent of omics technologies, electronic health records and other settings that lead to high-dimensional data, means that machine learning approaches to data analysis will become increasingly important in epidemiology. For this to be a successful approach to drawing causal inferences from data, the predictive modelling aspects (to be performed by the machine) must be separated from the subject matter considerations, such as the specification of the estimand of interest, and the encoding of plausible assumptions concerning the structure of the data-generating process (to be performed by humans). Whereas traditional epidemiological approaches to the analysis of data naturally blur the two aspects, the FACE makes the distinction explicit, and hence allows machine learning methods to be successfully employed.^33^

### An enabling or a paralysing approach?

Its emphasis on definitions and assumptions has sometimes given the false impression that the FACE is a ‘paralysing’ approach. How should the applied epidemiologist proceed in settings where clear definitions are hard and assumptions are violated, but nevertheless quantitative causal inference is needed? The advice that accompanies the theory is pragmatic, for example:

The more precise we get the higher the risk of nonpositivity in some subsets of the study population. In practice, we need a compromise.^34^

The emphasis is on adding to the statistical toolbox so that a greater range of questions can be addressed under less strict assumptions, and sensitivity analyses carried out so that appropriate transparency and scepticism enter the interpretation of results:

Methodology almost never perfectly corresponds to the complex phenomena that give rise to our data. Methodology within a field ought to advance in expanding the range of questions that can be addressed, in relaxing the assumptions required, and in allowing investigators to assess the sensitivity of conclusions to violations in the assumptions.^35^

### The focus of causal enquiries in epidemiology

We contrast two statements:

Statement 1: Exposure E is a cause of disease D.

Statement 2: The effect of exposure E on disease D, expressed as a risk ratio, comparing exposure level 1 vs 0, is 1.2, and this 20% increase in risk is (or is not) of sufficient magnitude to be scientifically meaningful.

Recalling the extensive discussions at the turn of this century on *P*-values vs confidence intervals,^36–38^ the consensus among the epidemiological community—probably more so than in any other scientific community—is that knowing whether or not an exposure causes a disease (Statement 1) is less important than knowing whether or not an exposure causes a disease to at least a minimally scientifically meaningful extent (Statement 2). To be able to judge whether a scientifically meaningful effect is attained, it should therefore be clear from the results of an epidemiological study: (i) what is the meaning of the exposure; and (ii) what effect size measure is being used. For example, to understand statements such as ‘weight loss which was unintentional or ill-defined was associated with excess risk of 22 to 39%’,^39^ one needs to understand the distribution of weight loss.

We believe that some of the apparent discrepancies between the philosophical and epidemiological standpoints on causality stem from a failure to acknowledge the difference between the two statements above, and the different levels of care and detail required when inferring such statements from data. It is well-known in many settings that effect estimation requires additional assumptions on top of what is required for testing the causal null hypothesis, for example methods that use instrumental variables.^40^

## Misconceptions about the FACE in VBP and KDS

There are three main shared misconceptions on which VBP and KDS build their arguments. We discuss each in turn below.

### Misconception 1: The dominant view in the FACE is that hypothetical interventions must be currently humanly feasible

This idea is central to much of VBP’s and KDS’s criticisms of the FACE, but we do not believe it to be a correct characterization of the dominant views within the field. The FACE advocates having in mind hypothetical interventions that are ideally (close to being) unambiguously defined, and this is what is evident from the quotations chosen by VBP. We do not agree with their deduction from these quotations (nor do we interpret from the opinions expressed in the field more generally) that these hypothetical interventions need be currently humanly feasible, except of course when the purpose of the investigation is to guide imminent practical policy decisions. The statement by VBP on page 6, ‘in order for an intervention to be well specified… it is not necessary that the intervention can be done; there is a difference between specifying and doing’, is uncontentious in our view. Sufficient specificity is the ideal, and not feasibility.

In spite of this, the work from the FACE makes explicit that the results from a causal analysis relate to all hypothetical interventions, whether feasible and/or unambiguously defined or not, that—as well as the usual conditional exchangeability assumptions—satisfy the so-called consistency assumption. This includes all hypothetical interventions which are non-invasive in the following sense: if they were applied to set the exposure to some value x for all subjects, they would not change the outcome in subjects who happen to have that exposure level x, from what was actually observed.

Furthermore, since consistency at an individual level can be relaxed to a slightly weaker version of the same assumption, Herna´n and VanderWeele^41^^,^^42^ show that it is possible to proceed even when a single non-invasive hypothetical intervention seems inconceivable, provided that a non-invasive ensemble of hypothetical interventions is conceivable. The exact form of this depends on the context but, for example, it is often consistency in expectation given confounders; i.e. that if a hypothetical intervention were applied to set the exposure to some value x for all subjects, this would not change the conditional expectation of the outcome given confounders in subjects who happen to have that exposure level x, from the conditional expectation of the observed outcome given confounders among these subjects with exposure level x. For example, in an observational study of the effects of obesity, the work by Herna´n and VanderWeele^41^ shows how the interpretation of any causal effect measure estimated from a typical observational study pertains (under all other relevant assumptions) to a stochastic complex hypothetical intervention that shifts the distribution of many different obesity-related exposures. Knowledge about the effects of such a hypothetical intervention is of limited value for immediate practical policy decisions, but is relevant for scientific understanding.

A growing body of work from the FACE is therefore focused on epidemiologically important exposures for which certainly no humanly feasible intervention is known, and often no single non-invasive hypothetical intervention could be conceived of for which the observational data are informative. For example, Bekaert *et al.*^43^ investigate the impact of hospital-acquired infection on mortality in critically ill patients, with the aim of estimating the intensive care unit mortality risk that would have been observed had all such infections been avoided. Their analysis aims to give insight on how harmful these infections are, even though no feasible intervention exists that could prevent infection for all. By the consistency assumption, the authors view their results as being informative about the net effect of infection. This effect may differ from the effect of an intervention to prevent infection, which—if it could be designed—would likely do more than just prevent infection. Other exposures that have been recently studied in this context are, for example, socioeconomic position, delirium in critically ill patients, weight change, viral clearance and depression.^44–50^

Petersen and van der Laan^51^ discuss the feasibility and specificity issue in a recent overview of the FACE, stating that:

There is nothing in the structural causal model framework that requires the intervention to correspond to a feasible experiment … if, in addition to the causal assumptions needed for identifiability, the investigator is willing to assume that the intervention used to define the counterfactuals corresponds to a conceivable and well-defined intervention in the real world, interpretation can be further expanded to include an estimate of the impact that would be observed if that intervention were to be implemented in practice.

Much of the recent work stemming from the FACE has been dedicated to the study of mediation,^9^ in particular using so-called natural direct and indirect effects. These effects have been criticized by some^52^ precisely because they concern hypothetical interventions that are, by their very definition, humanly unfeasible (irrespective of the variables being studied); in other words, no randomized experiment could even in principle be constructed that would allow the estimation of these effects under assumptions guaranteed to hold by design. The dominant view within the FACE is that these effects, because of the importance of the epidemiological questions they aim to address, are worthy of our attention despite the very strong unfeasibility of the hypothetical interventions they demand be imagined.

### Misconception 2: The FACE sees the RCT as the best choice of study design for causal inference

In order to dispel this misconception, we start by proposing what we believe the characteristics of the ideal study to be, when inference about the total effect of a single (time-fixed) exposure is the goal. By ‘ideal’ we mean the study we would run if our concerns were only scientific, with no regard whatsoever for practicality, ethics or cost. We believe that such a study would have (at least) the following characteristics (and many more, of course):

- no inclusion/exclusion criteria [so that the effect of the exposure in a variety of different groups can be separately estimated, as well as standardized effects to different (sub-)populations if relevant];
- large sample size (also thereby ensuring a large number of events if relevant);
- an unambiguously defined set of levels for the exposure (often more than two if dose–response is of interest) allocated at random;
- long follow-up (so that short-, medium- and long-term effects can all be separately estimated);
- rich baseline covariate data (so that effect modification can be explored);
- and no attrition, other forms of missing data, noncompliance or measurement error.

It is true that point (iii) says that the ideal study would be randomized (hence the fact that the FACE often talks of ‘the idealised randomized experiment’), but does this imply that realistic RCTs are necessarily to be viewed as better than realistic observational studies for causal inference? No; because observational studies in practice are more likely to get closer to points (i), (ii), (iv) and often also (v). The ideal study, which has as one of its characteristics that it is randomized, is in some respects closer to a realistic RCT and in other ways closer to a realistic observational study. Only by knowing the specific context can a judgement be made on which is better for that context, if indeed both are feasible, ethical and practical. In many settings, when a RCT would be unfeasible, the FACE advocates having in mind the ideal (randomized) study, merely as a mental device to ensure that the observational study is designed and analysed in the most sensible fashion. This is even more valuable in complex longitudinal studies such as those that attempt to determine the optimal dynamic decision strategy.^53^^,^^54^

Since a key difference between a realistic observational study and the ideal study above is that (iii) doesn’t hold, a major focus of the methods arising from the FACE is how the realistic observational study can be analysed in such a way that it emulates the ideal study with respect to (iii). This does not equate to the view that the FACE strives to analyse realistic observational studies in such a way that the results obtained are close to those that would have been obtained from a realistic RCT on the same exposure. The ultimate aim is to analyse realistic observational studies in such a way that the results obtained are close to those that would have been obtained from the ideal study, one feature of which is that the exposure is randomized. These two aims are different, and an investigation of this difference led to important insights regarding the hormone replacement therapy (HRT) controversy by Herna´n *et al.*^55^ Taken out of context, the title of the article by Herna´n *et al.* ‘Observational studies analyzed like randomized experiments’ could wrongly be taken to strengthen this misconception, that:

Proponents of [the FACE] assume and promote the pre-eminence of the randomized controlled trial (RCT) for assessing causality; other study designs (i.e. observational studies) are then only considered valid and relevant to the extent that they emulate RCTs. [VBP, page 2]

On the contrary, Herna´n *et al.* were not advocating that observational studies should be analysed like randomized experiments. Note that the same lead authors have written articles with the following titles: ‘Randomized trials analyzed like observational studies’^56^ and ‘Observational studies analyzed like randomized trials, and vice versa’.^57^ Herna´n *et al.* dropped many years of follow-up from their data, together with many subjects who would not have met the trial’s eligibility criteria, and ignored the information they had on treatment discontinuation, in order to emulate the intent-to-treat analysis performed in the RCT: it would be madness to advocate any of these measures as the best analysis of the observational data. Rather, Herna´n *et al*'s aim was merely to show that if one did analyse the observational study so as closely to mimic a randomized trial, the contradiction between the results from the RCT and observational studies would be nearly eliminated.This served to challenge the dominant view at the time that the contradiction was due to unmeasured confounding in the observational studies. Incidentally, this work by Herna´n *et al.* on the HRT controversy is an example of hypothesis elimination, as advocated by VBP and KDS. As further evidence that this misconception is unfounded, we refer here to the large body of work from the FACE on the analysis of data from retrospective study designs (e.g. case–control studies).^58–71^

### Misconception 3: The FACE believes that sex, race and genes can’t be causes; furthermore (in KDS) that racism can’t be a cause

#### Sex, race, sexism and racism as causes

This issue, particularly with respect to race, has been the source of recent controversy^72^ in part in response to VanderWeele and Herna´n,^73^ and VanderWeele and Robinson.^74^ We see this controversy (‘is race a cause’?) as something of a storm in a teacup as far as epidemiology is concerned, brought about perhaps by the different focuses that philosophers and epidemiologists have when it comes to causality (note that both Glymour and Glymour^72^ and VBP, which has two joint lead authors, have philosophers as lead authors, and KDS also refer extensively to the philosophical literature on causality). Referring back to Statements 1 and 2 given earlier, philosophers tend to concern themselves with the meaning of statements of type 1, whereas epidemiologists are more concerned with statements of type 2 and—very importantly—whether or not it is justified to make a statement such as statement 2 from the data at hand. It would be very strange to claim that sex and race cannot be considered in place of E in Statement 1. However, using them in place of E in Statement 2 requires some care.

It is the dominant view within the FACE (and we agree) that asserting that ‘this group of Caucasians would have had a 20% lower risk of disease D had they been Afro-Caribbean’ is meaningful only if the statement’s readers share a near to common understanding of what ‘had they been Afro-Caribbean’ means, and evidently this requires further details. In the counterfactual world are they to be Afro-Caribbean from conception? And in what sense? Are their genes hypothetically being switched for genes that are drawn from the distribution of genes seen in Afro-Caribbeans? Are they to be brought up in their biological Caucasian families, or similar Afro-Caribbean families? What constitutes similar? Again, the consistency (and conditional exchangeability) assumption rules out many (or all) of the above hypothetical interventions. In order to understand which, further details must be specified, for example whether the Afro-Caribbean study participants were brought up in biological Caucasian families or not.

Why do we think that this is a storm in a teacup? Because epidemiologists are rarely interested in what would have happened to these males had they been females, nor in what would have happened to these Caucasians had they been Afro-Caribbeans; rather, they are interested in one of three possible things: (i) sex and race as effect modifiers; (ii) describing gender and ethnic inequalities, and then in seeing what can be done to reduce them which, as VanderWeele and Robinson show, can be done without needing to define hypothetical interventions on sex/gender/race/ethnicity; or (iii) the effect of the perception of race and sex, that is in the effect of racism and sexism; this is what KDS talk about in their third example. None of these requires defining hypothetical interventions on sex/gender/race/ethnicity. For (iii), the hypothetical intervention would be on the perception of race/sex, rather than on race/sex itself.^75^

We stress that the FACE is not saying that studying sex and race is not important; evidently these factors are central to many important epidemiological research questions. The ‘alarm’ that KDS feel follows precisely from the confusion that ensues when causal inference is too informally discussed; they have misconstrued the observation made by the FACE that it is difficult to answer the question of ‘what would happen if we changed sex/race’ and that in any case we are more likely interested in one of (i), (ii) or (iii) above, as saying that we should not study sex and race (or even sexism and racism) at all. They write, ‘One alarming feature of [the FACE] is the re-appearance of previously rebutted causal claims that ‘race’ [. . .] cannot be a ‘cause’ because it is not ‘modifiable’’, before going on to explain that it is the effect or racism, rather than the effect of race, that is of interest to them.

It can be seen from the applied literature on investigations of ethnicity, for example, that these investigations are indeed described using associational (not causal) language, for example:

Māori and Pacific infants were twice as likely as European infants to have a mother who was obese … ethnic differences in overweight were less pronounced.^76^

The same is seen when sex/gender is studied. For example, in the recently published UK Chief Medical Officers’ guidelines on safe alcohol drinking,^77^ gender played a key role. The committee of experts reviewed a large body of evidence on the causal effect of alcohol consumption on health outcomes, in men and women separately, and concluded that the guidelines on safe consumption limits should be the same for both genders. This was based on a study of effect modification by gender.^78^ Such effect modification is associational with respect to gender (but causal with respect to alcohol consumption). The pertinent question in this context did not therefore require imagining hypothetical interventions on gender.

#### States, including genes, as causes

VBP discuss the FACE’s view of statements such a ‘100 000 deaths annually are attributable to obesity’ and correctly characterize one of the FACE’s objections to this statement as stemming from its vagueness. The statement implies something along the lines of *had there been no obesity*, there would have been 100 000 fewer deaths annually, **or* were we hypothetically to eradicate obesity*, there would be 100 000 fewer deaths annually. As discussed by Herna´n and Taubman,^79^ the words in italics are ambiguous; for example have those who have hypothetically lost weight lost weight from their waist, or their hips or both, and if so in what combination? Current evidence from cardiovascular epidemiology suggests that the consequences of these different possibilities would be different. Once more, the consistency assumption helps to resolve this ambiguity, but understanding its implications requires a detailed appreciation of the distribution of obesity-related exposures in the study population, as discussed by Herna´n and VanderWeele.^41^^,^^42^

What is relevant to the current misconception, in particular in relation to genes as exposures, is the following characterization of the FACE given by VBP on page 6. They extrapolate from the issue concerning obesity and conclude that under the precepts of the FACE:

‘States’ like obesity (or hypercholesterolaemia, hypertension, carrying BRCA1 or BRCA2, male gender) can no longer be seen as causes.

Thus, they have concluded that the FACE believes that the causal effects of genes (along with many other things) cannot be studied. We strongly oppose this conclusion. Hypothetical interventions on body mass index (BMI) are too ambiguous (to imagine an obese person as not obese, there are many other changes that need also be imagined, and a myriad possibility for these) unless one elaborates further. However, the idea that a mutation in the BRCA1 gene inherited at meiosis could instead hypothetically not have been inherited, although currently unfeasible to implement, is sufficiently well-specified. This is so in the sense that imagining that all other inherited genes and all environmental conditions at the time of meiosis remain the same as in the actual world, would reasonably suffice for the hypothetical intervention to be non-invasive. There are many instances in the key texts cited by VBP, KDS and beyond where the causal effects of genetic variants are discussed by the FACE.^67^^,^^69^^,^^80–84^

## Further misconceptions in KDS about the role of DAGs in causal inference

The description by KDS of the role played by DAGs in causal inference is counter to what is written in the key textbooks and papers in this area, and counter to what is taught in introductory courses to causal inference. We start, therefore, by clarifying the role of DAGs in causal inference, before pointing out the key misconception that underlies many of KDS’s criticisms. We end this section by pointing out further errors in their discussion of the DAGs relating to their three examples.

### DAGs in statistics

As used generally in statistics, DAGs are pictorial representations of conditional independences. The absence of an arrow between two nodes in a DAG is used to represent conditional independence between the two variables represented by these two nodes, conditional on the variables represented by the nodes’ parents in the graph; let us call these conditional independences ‘local’. The advantage of representing local conditional independences graphically is that ‘global’ conditional independence statements (i.e. conditional independences between two variables given sets other than those represented by the nodes’ parents in the graph) can be deduced from the local conditional independences used to construct the graph, via an algorithm known as d-separation.^85^

### DAGs in causal inference

DAGs are appealing for causal inference since the causal effects of interest can be characterized in terms of specific conditional dependencies between exposure and outcome. DAGs provide insight as to which conditional dependences characterize the effect of interest, by elucidating the causal structures that would render exposure and outcome conditionally dependent. Causal structures are here implied by the data-generating mechanism, which involves information on the direction of causal effects, the absence of common causes between variables, the absence of direct effects between variables and study design. Such information, which is not contained in the data but may be available from subject-matter knowledge, can be encoded in the causal DAG.

The DAGs used in causal inference can be interrogated (using d-separation, after some slight manipulation, e.g. removing arrows emanating from exposure, or constructing the corresponding single world intervention graph (SWIG)) to see if, for example, a given set of variables is sufficient to adjust for confounding given the assumptions encoded in the causal DAG. DAGs have thus proved very useful in this process since humans are well-known to have poor probabilistic intuition about the consequences of conditioning or adjusting. By explicitly visualizing the consequences of conditioning, DAGs help to circumvent the intuitive errors that might happen when this process is attempted informally.

We stress that the DAGs used in causal inference express a priori knowledge and hypotheses; see, for example, the paper by Robins^86^ in which he shows how identical data can be analysed in different ways, when guided by different causal DAGs, according to the different possible study designs, questions of interest, and subject matter knowledge that underpin/accompany these data.

### Misconceptions regarding DAGs in KDS

In the light of the above clarifications, it is now possible to address KDS’s criticisms of DAGs. They point out many times that data alone are not sufficient to arrive at the DAG nor at causal inferences (‘data never speak by themselves’). This is indisputable, and is precisely why DAGs are useful in causal inference: to make the assumptions based on a priori knowledge explicit, and to facilitate the translation of a priori knowledge into a suitable statistical analysis. They write that ‘there is no short cut for hard thinking about the biological and social realities and processes that jointly create the phenomena we epidemiologists seek to explain’, and we agree. Causal DAGs don’t purport to provide such a short cut; the causal DAG is the result of the hard thinking, not a substitute for it, and the short cut provided is via d-separation, which enters the next step in helping the transition from the result of this hard thinking to a sensible statistical analysis. Many of their criticisms are along similar lines and follow from the same underlying confusion, for example when they write, ‘Nor can a DAG provide insight into what omitted variables might be important’. We agree of course: it is the background knowledge that leads to the DAG, and not vice versa.

On page 9, KDS indicate that the world is too complicated to hope to understand all the relevant causes of the exposure in question (‘one would need infinite knowledge, after all, to generate an exhaustive list’) and we, once more, agree. However, the many examples from the FACE have demonstrated that even when the DAGs are unavoidably simplistic, they do provide much insight into the biases inherent in certain statistical analyses.^87^

### KDS’s examples

We found the discussion by KDS of their three examples rather difficult to follow, precisely since the DAGs they allude to are not drawn. This in itself points to the usefulness of DAGs for clarity of thought and communication in these settings.

#### Example 1: Pellagra

**Figure 1.**
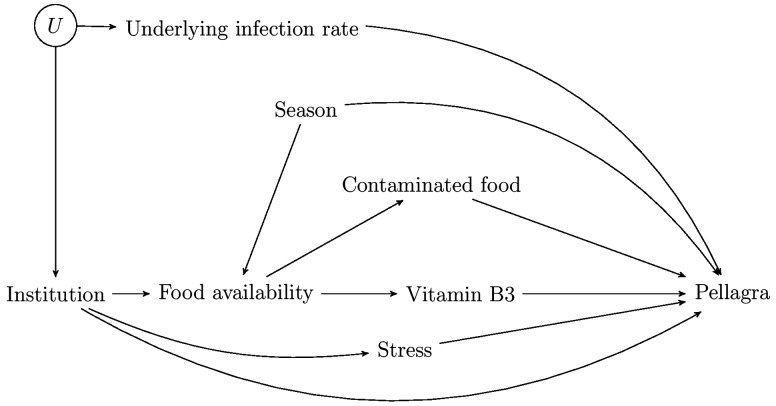
A casual DAG representing all the hypotheses discussed by KDS in relation to the effect of institutionalisation on pellagra infection.

In Figure 1, we have drawn a DAG capturing KDS’s discussion of the pellagra example. KDS describe the two leading hypotheses (germs and contaminated food) as containing the same elements but with arrows ‘that pointed in entirely opposite directions’. We don’t believe this to correspond to their description nor to the plausible relationships involved. In the ‘germ theory’, those with a high infection rate were believed to be more likely to be institutionalized, but it would not be plausible that the infection caused institutionalization; rather, both would share common causes (depicted by U in our diagram) such as poverty (and hence the capitalism hypothesis is also depicted). In the remaining hypotheses they describe, there is a causal effect of institutionalization on pellagra infection, but via different potential mediators: contaminated food, stress and vitamin B3 deficiency. Each hypothesis introduces a new element(s) into the DAG and all can be depicted in a single DAG, as we have done in Figure 1; no reversal of any arrows is involved. Of course, subject matter knowledge is needed to reach the DAG, and data analysis is then required to evaluate which are the strongest pathways, in order to determine which hypothesis (or hypotheses) is correct. The DAG in isolation is insufficient for arriving at an explanation (or for ‘alone wagging the causal tale’), of course, but we are unaware of claims to the contrary.

#### Example 2: Birthweight paradox

**Figure 2.**
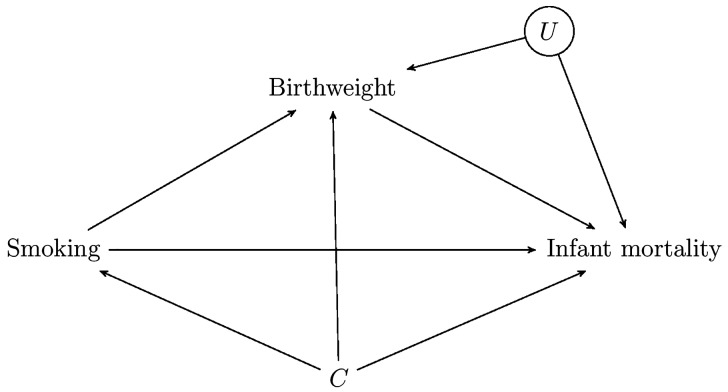
A casual DAG for the ‘birthweight paradox’.

Figure 2, which is the DAG alluded to by KDS in reference to the birthweight paradox, shows that, even if we had measured and adjusted for all confounders C of smoking and infant mortality, as long as there exist unmeasured common causes U of birthweight and infant mortality, then a comparison of the mortality rates of low birthweight babies between smoking and non-smoking mothers does not have a causal interpretation. This is because stratifying on birthweight induces a correlation between smoking and U, in such a direction that it could explain the paradox. As VanderWeele writes in a recent review article on this issue:^88^

The intuition behind this explanation is that low birthweight might be due to a number of causes: one of these might be maternal smoking, another might be instances of malnutrition or a birth defect. If we consider the low birthweight infants whose mothers smoke, then it is likely that smoking is the cause of low birthweight. If we consider the low birthweight infants whose mothers do not smoke, then we know maternal smoking is ruled out as a cause for low birthweight, so that there must have been some other cause, possibly something such as malnutrition or a birth defect, the consequences of which for infant mortality are much worse. By not controlling for the common causes (U) of low birthweight and infant mortality, we are essentially setting up an unfair comparison between the smoking and non-smoking mothers. If we could control for such common causes, the paradoxical associations might go away.

VanderWeele chooses malnutrition and birth defects as possible Us, whereas KDS choose ‘harms during their fetal development unrelated to and much worse than those imposed by smoking, e.g. stochastic semi-disasters that knock down birthweight as a result of random genetic or epigenetic abnormalities affecting the sperm or egg prior to conception or arising during fertilization and embryogenesis’. Is this not just a biologically more detailed description of the sort of phenomenon involved in the development of a birth defect, in which malnutrition could also play a part? In other words, the ‘DAG explanation’ and KDS’s explanation are almost the same, and indeed, since the ‘DAG explanation’ only posits that such a U may exist, it subsumes KDS’s slightly more detailed explanation. We don’t understand their claim, therefore, that the former explanation is incorrect, while the latter is ‘lovely’.

Their comment that, having identified the potential for collider bias in a DAG, ‘it is another matter entirely, however, to elucidate empirically, whether the hypothesized biases do indeed exist and if they are sufficient to generate the observed associations’ is of course entirely uncontentious. This is precisely why, having identified the possibility that the paradox could be explained in this way, the FACE went on to evaluate whether or not plausible magnitudes for the effects of such U on birthweight and infant mortality would suffice to explain the reported paradoxical associations.^25^^,^^89^^,^^90^

In summary, DAGs are neither the beginning (they arise from subject matter knowledge) nor the end (they guide the subsequent data analysis and/or sensitivity analyses), but neither has the FACE made claims to this effect.

#### Example 3: Racism

As we discussed under Misconception 3 above, KDS are in agreement with the FACE in their discussion of their third example, since hypothetical interventions on racism don’t suffer from any of the specification problems that accompany hypothetical interventions on race discussed above and in the literature that they criticize. Rather than saying that the FACE is ‘bark[ing] up the wrong tree, and indeed miss[ing] the forest entirely’, KDS should surely aim this criticism at their fellow critics of the FACE, such as VBP, who are the ones advocating studying the causal effects of race and sex; the FACE has merely outlined the difficulties in doing so, and entirely agrees that it is unlikely to be the true question of interest.

## Discussion

### Formality and non-invasive hypothetical interventions

In view of the difficulties of making causal enquiries based on observational data, epidemiologists have historically tended to speak only of associations. VBP rightly say that the FACE has been a response to this ‘retreat to the associational haven’. Although prudence is imperative, incidentally, this ‘retreat’ has tended to result in a lack of prudence in data analysis. Indeed, since essentially all statistical analyses are designed to measure associations, adjusted or not, the lack of a formal framework makes it impossible to distinguish clearly between analysis strategies that target the envisaged causal enquiry from those that do not. The unfortunate result has been reflected in analysis strategies that tend to induce bias, even in the ideal setting where all relevant confounding variables are perfectly measured.^4–7^

To be able to identify, from across the many possible associations between exposure and outcome that one could measure, the one that targets the causal enquiry at stake, the FACE has adopted the notion of hypothetical interventions. Using such hypothetical interventions, effect measures of interest can be clearly expressed, identifying assumptions can be explicated and analysis strategies developed that are valid when these assumptions are met. The FACE thus merely aims to provide a principled framework under which causal enquiries can be approached. It does not eschew the many sources of epidemiological information, such as time trend data, retrospective designs, negative controls etc., but rather aims to understand under what conditions such information enables causal enquiries to be answered; there are examples of this work by the FACE in relation to time trend data and negative controls.^91–97^ In addition, it aims to caution epidemiologists that a good understanding of a reported effect requires a specific understanding of the exposure and considered effect measure.

Adopting the specific interventionist framework as a philosophy, we have argued that the formality that underlies the FACE does not require the existence of humanly feasible interventions, as it targets ‘non-invasive interventions’ in the sense implied by the consistency assumption. We believe that many epidemiological enquiries, except those that aim to evaluate the impact of public health interventions, implicitly have such interventions in mind.

### Alternative frameworks

A number of causal theories have attempted to move away from the mainstream approach as described above, by not using potential outcomes.^99–101^ Some of these, in particular the decision-theoretical framework, have been useful in highlighting some strong assumptions entailed in approaches based on potential outcomes, particularly when joint or nested counterfactuals are involved. The decision-theoretical framework adheres to the same principles (one might argue even more strongly) of clearly expressing the causal target of estimation and the assumptions under which this can be identified. Indeed, in terms of data analysis, the decision-theoretical approach reproduces existing results from the potential outcomes approach, and we view it as a part of the FACE. Other causal theories, in their attempt to avoid potential outcomes, have tended to be less explicit, thereby obscuring and eventually ignoring certain selection biases. VBP and KDS similarly recommend that other philosophical frameworks for causality be adopted in epidemiology. We hope that their alternatives, which are not sufficiently specific to be fully evaluated, will not run into the same difficulties.

Both VBP and KDS mention the need for the synthesis of evidence across multiple studies and settings. We agree with this, and view the concepts and methods of the FACE as aiding rather than impeding this endeavour, in two ways: (i) more reliable causal analyses of the individual studies contributing to a synthesis improves the reliability of the synthesized conclusion; and (ii) by being clear what question is being addressed, and under what assumptions the analysis strategy used can be deemed successful, evidence from different studies can be more reliably combined. We cite a recent example of where a meta-analysis came to suspect conclusions based on shortcomings in both these aspects.^102^

VBP and KDS suggest the analysis of time trend data, the use of negative controls and the elimination of alternative hypotheses, but as we have discussed, these are already done within the FACE.^91–97^ Arguably, the vast section of the FACE literature dedicated to sensitivity analyses has at its core the elimination (or at least consideration or evaluation) of alternative hypotheses. A novel approach to the elimination of alternative hypotheses is described by Rosenbaum.^98^ VBP also imply that Pearl’s framework [specifically non-parametric structural equation models (NPSEM)]^85^ is more amenable to epidemiological enquiries. Whereas of course we view the NPSEM framework as belonging to the FACE, it is well-known that the NPSEM framework is more demanding in terms of the assumptions it makes than alternative frameworks within the FACE.^103^ These are specifically assumptions similar to consistency. Instead of making the consistency assumption only with respect to hypothetical interventions on the exposure, the NPSEM assumptions imply consistency with respect to hypothetical interventions on every variable in the causal diagram. We fail to follow therefore why VBP might be prepared to accept this more restrictive sub-framework while viewing the larger framework that contains it as too restrictive.

### Historical success stories

Both VBP and KDS draw attention to a few historical examples from epidemiology’s past in which successful causal inferences were achieved without the formality advocated by the FACE. We should be cautious of basing future strategy on these ‘cherry-picked’ success stories, without mentioning the numerous failures. Indeed, a similar reasoning would lead one to conclude that science does not need a formal deductive theory at all, since there are obviously many examples, e.g. in prehistoric times, where science and knowledge acquisition progressed without formal theories. The logical error in this reasoning is that no consideration is given to the many examples where plain intuition and informal deduction have been misleading. This does not mean that informal approaches have no value; they should and do guide the design of studies and statistical analysis, but objective science eventually calls for a formal theory and approach.

We view the FACE as precisely offering formal tools to investigate cause–effect relationships. They are always guided by what KDS call IBE (inference to the best explanation). Indeed, IBE is often how one comes to investigate the specific cause–effect relationship in the first place. Given how associations can be distorted in complicated ways due to implicit/explicit conditioning or not conditioning, and how intuition, for example in mediation analysis and instrumental variable methods, breaks down as soon as nonlinear relationships are at play, there is no question in our opinion that a formal theory is needed to guide data analysis.

### Concluding thoughts

Throughout its history, aspects of the FACE have been misconceived by some. Its tendency to be explicit about assumptions has often been misunderstood as if this framework needs more assumptions than traditional alternatives. This has then led people to use ‘associational analyses’ instead, the conclusions from which they eventually interpret causally, where causal interpretation is only justified under even stronger assumptions.

These papers by VBP and KDS highlight further misconceptions which, if true, would mean that many important exposures would be excluded from being studied within the FACE framework and many tools, such as causal DAGs, rejected as misleading. In this response, we have attempted to correct these misconceptions and, while stressing the clarity that comes from having a rigorous framework based on clear definitions and assumptions, we have highlighted the pragmatic considerations that should and do accompany the theory when applied in practice, together with the central role played by subject matter knowledge. We are glad to learn about these concerns, and to be able to clarify that the FACE does not refute epidemiological questions that cannot be linked to humanly feasible interventions, nor epidemiological designs that cannot emulate aspects of randomized studies, and nor does it claim that graphical or statistical methods lessen the importance of subject matter knowledge. Rather, the FACE aims to provide insight on what can be learned about these questions and from these designs under the most plausible assumptions possible, given the data, design and subject matter knowledge at hand.

As Herna´n^104^ concluded in a recent debate on similar issues, relating to whether or not left-truncated data can meaningfully be used in causal inference:

Exceptions to this synchronizing of the start of follow-up and the treatment strategies may be considered when the only available data (or the only data that we can afford) are left truncated. If we believe that analyzing those data will improve the existing evidence for decision-making, we must defend the use of left-truncated data explicitly, rather than defaulting into using the data without any justification.

We understand from this, and agree, that no data and no questions are ‘off limits’ as long as the data are informative about the question. The core theme of the FACE is that formality allows one to assess to what extent the data at hand are informative about a particular question given subject matter knowledge. A rejection of this framework in favour of an alternative would either mean that the new framework could do away with the need to link the data to the question, or that the required link would remain but in an obscured and less explicit fashion. The former would be miraculous, and the latter would increase the risk of confusion and misinterpretation.

## Supplementary Data

Supplementary data are available at *IJE* online.

## Funding

R.D. is supported by a Sir Henry Dale Fellowship jointly funded by the Wellcome Trust and the Royal Society (grant number 107617/Z/15/Z). The LSHTM Centre for Statistical Methodology is supported by the Wellcome Trust Institutional Strategic Support Fund, 097834/Z/11/B. S.V. acknowledges support from IAP research network grant no. P07/06 from the Belgian government (Belgian Science Policy).

## Supplementary Material

Supplementary Data
