# Supplementary material for: Commentary: The formal approach to quantitative causal inference in epidemiology: misguided or misrepresented?
Source: Int J Epidemiol. 2017 Jan 27;45(6):1817–29. doi: 10.1093/ije/dyw227 (PMC5841837; doi:10.1093/ije/dyw227)
Supplement: Supplementary Data [file dyw227_supp.pdf]

Supplementary Material

The approach advocated by the FACE applied to an investigation of urinary tract infections and low birthweight

Suppose we are interested in the potential causal relationship between urinary tract infections (UTIs) in the 2nd and 3rd trimesters of pregnancy and the subsequent low birthweight (LBW) of newborn babies, and that existing epidemiological evidence is suggestive of a clinically meaningful causal effect but not definitively so. Thus, a large prospective cohort study is designed, with laboratory assessment of the exposure in the second and third trimester of pregnancy.

In this appendix, we will outline how some of the key concepts from the FACE might be applied to the analysis of this cohort study and the interpretation of the results. We are not aiming to cover all possible concerns here; of course in any given real example there will always be specific additional features and complexities to be considered. Rather, we aim to highlight the thinking and the assumptions as they relate to a particular simple example, and above all to outline how the linking of the target causal estimand and the data can be achieved via these assumptions in a few simple mathematical steps. We recommend that those wanting to read about more realistic examples, with numerous additional complexities, turn to the many substantive papers cited in the main manuscript, *e.g.* [10–13, 43].

As with any epidemiological investigation, we would start (at the point of designing the study, more than when starting the analysis) with establishing what exactly is the question of interest. This involves asking:

A. What is the population of interest? All pregnant women living in the particular region/country to be studied? Perhaps non-singleton births should be excluded? And so on.

B. What exactly is the exposure of interest? Are all subtypes of UTIs to be included? What about those experiencing more than one UTI during pregnancy? What about the timing of acquiring the UTI(s)? *etc.*

C. How will the outcome be classified in pre-term births? *etc.*

D. Do we simply want to quantify the magnitude of the effect of maternal UTIs on the prevalence of LBW in the population of interest? Or are we interested in particular subgroups, *e.g.* particular racial groups, or mothers with gestational diabetes? Are we interested in investigating possible synergistic effects of other infections? And so on.

Let us assume for simplicity that the population of interest is all full-term singleton live-born babies from a particular region in a particular period, and that the exposure is simplified to ever having experienced a UTI in the second and/or third trimester of pregnancy versus never in these two trimesters of pregnancy. Let X be the binary exposure (maternal UTI, ever, coded 1, or never, coded 0), Y the binary outcome (LBW newborn, yes or no) and $\mathbf{C} = C_1, \dots, C_p$ a set of potential confounders, *e.g.* maternal age, comorbidities (such as diabetes), other maternal infections and socio-economic factors.

The following features would characterise a FACE approach to this example:

1. **Estimand**: Express the question (or questions) of interest mathematically, *i.e.* as an estimand (or as estimands).

Potential Outcomes
Since causal questions involve not just features of the data at hand, but also a notion of *how things would have been had something been different*, then expressing causal questions mathematically requires an extension to the traditional statistical language of expectations, variances, probabilities, odds, *etc.* The extension most commonly used in the FACE is that of potential outcomes.

For each infant in the population of interest, let $Y(0)$ denote the outcome that would have been observed for that infant had the mother, possibly counter to fact, not been exposed during pregnancy, and let $Y(1)$ denote the outcome that would have been observed had the mother been exposed.

Possible Estimands
A population-level causal effect is then expressed as a contrast between the distributions of $Y(0)$ and $Y(1)$. For example, a marginal causal risk difference is:

$$E\left\{Y(1) - Y(0)\right\}$$

where expectations are taken over the population of interest. This estimand is interpreted as the difference in prevelance of LBW if, hypothetically, all pregnant women would be exposed, versus if no pregnant woman were exposed.

If we were interested in effect modification by maternal age, then we could compare the following conditional risk difference:

$$E\left\{Y(1) - Y(0)|A = a\right\}$$

for different values of a, where A is the age of the mother.

Suppose a different type of maternal infection, MIB, together with UTI, were believed to have a possible synergistic effect on LBW, then we would redefine our potential outcomes to include MIB too. $Y(0,0)$, $Y(1,0)$, $Y(0,1)$ and $Y(1,1)$ could be used to denote, respectively, the potential value that Y would take were both infections to be absent, only UTI present, only MIB present, or both exposures present. A synergistic effect of the two exposures would be present if:

$$E\left\{Y(1,1) - Y(0,1)\right\} > E\left\{Y(1,0) - Y(0,0)\right\}$$

i.e. if the effect of UTI is more pronounced when combined with MIB.

The estimand can also be chosen so as to reflect an interest in the excess prevalence of LBW that is attributed to UTIs, *i.e.*

$$E\left\{Y - Y(0)\right\}. \tag{1}$$

The possibilities are endless, but the resulting considerations are broadly similar, and so, for simplicity, in what follows, we assume that it is this final estimand that is of interest, *i.e.* the difference between the actual prevalence of LBW and the hypothetical prevalence of LBW that would be seen if UTIs could be prevented for all pregnant woman in the relevant population of interest.

Specificity
As we discuss in the main manuscript, for this estimand to be well-understood, the potential

outcome involved in it should ideally be close to being well-specified. This is not too problematic with an exposure such as infection. The hypothetical world in which no pregnant woman is infected could be one in which a hypothetical preventative treatment exists, with no (good or bad) side-effects, and is given to all pregnant women. It may also be useful to add that this hypothetical treatment be free for all pregnant women, so that in the hypothetical world in which all pregnant women are vaccinated, there are no knock-on effects on, say, their diet, due to having re-allocated resources towards acquiring the treatment.

For our chosen estimand, sufficiently well-specified $Y(0)$ would suffice. However, had we chosen an alternative estimand, we would also need to consider the meaning of $Y(1)$, and this could perhaps be a little trickier in this setting, since there could be many different reasons for acquiring a UTI present in the observed data. For simplicity, suppose there are two reasons: (1) high glucose level (*e.g.* due to poorly-controlled or undiagnosed diabetes), (2) incomplete voiding of the bladder. It may be plausible (from subject-matter knowledge) that the effect of maternal UTI on infant birthweight is the same regardless of the reason for its contraction, in which case the intervention (contract UTI by some means) would be sufficiently well-specified. However, if this were not the case, for example, if the UTIs acquired due to reason (1) were more severe than those acquired due to reason (2), then (for reasons that will become apparent below) the hypothetical intervention that we should imagine as giving rise to $Y(1)$ is a compound intervention (see [41]) in which the infection is contracted for reason (1) with probability π and for reason (2) with probability $1 - \pi$, where π is the proportion in the observed data who actually contracted the infection due to reason (1). To be as specific as possible, it would be good to try to infer π from the data. If this is not possible, then the ambiguity should be made explicit.

2. **Assumptions**: Depending on the analytic approach to be taken, different assumptions may be invoked. For example, instrumental variable analyses rely on a different set of assumptions than analyses based on confounder adjustment. Suppose, for example, that we will proceed by confounder adjustment, then the assumptions under which the causal estimand (1) above can be identified (*i.e.* re-written as functions of the distribution of the observed data) are:

   (a) The *consistency* assumption, which for this example states that:

   $$Y = Y(0) \text{ if } X = 0.$$

   In words, this says that for infants whose mothers were in reality not exposed, their outcome in reality is equal to what it would have been in the hypothetical world in which no mother was exposed. For this assumption to hold, the observed data need to be *relevant* for the hypothetical intervention we have in mind that gives rise to $Y(0)$. Or, in other words, the hypothetical intervention we have in mind should be non-invasive in the sense described in the main manuscript: the hypothetical intervention, when applied to prevent UTIs, should not change the outcome for pregnant women who would in reality not have been exposed, from the outcome that was in fact observed. Recall the caveats above that the hypothetical treatment should have no (good or bad) side effects and should be free. These caveats help to make the consistency assumption more plausible; they precisely concern the non-invasiveness of the hypothetical intervention.

(b) The *conditional exchangeability* assumption, which for this example states that:

$$Y(0) \perp\!\!\!\perp X | \mathbf{C}.$$

This states that, having adjusted for the observed set of potential confounders $\mathbf{C}$, the exposure and the potential outcome $Y(0)$ should be independent. In this example, $Y(0)$ denotes whether or not an infant would have been LBW had his/her mother, possibly counter to fact, not been exposed. It can therefore be viewed as a relevant summary of *all the other* determinants of LBW (observed or unobserved) apart from UTIs. If the exposure were to be associated with these other determinants, even after conditioning on all the measured confounders, this would mean that there is residual confounding, *i.e.* even after allowing for all the measured confounders, if UTIs are, say, more common amongst those with a higher risk of LBW even in the absence of UTIs, then adjusting for the measured confounders will not lead us to a causally-interpretable estimate of (1).

Causal diagrams are often advocated as a tool to help us decide how plausible the conditional exchangeability assumption is, based on existing knowledge (or plausible conjectures) regarding the causal structure of X, Y and $\mathbf{C}$. The more complex the situation (*e.g.* when variables contained in $\mathbf{C}$ are not simply common causes of X and Y) the more useful the causal diagrams are.

(c) The *positivity* assumption. For the case of our estimand, the relevant version is that, if all confounders $\mathbf{C}$ are discrete:

$$\text{For all } \mathbf{c} \text{ such that } P\{\mathbf{C} = \mathbf{c}\} > 0,$$
$$P(X = 0 | \mathbf{C} = \mathbf{c}) > 0.$$

In words, this says that, for a sufficiently large sample size, there should be unexposed mothers observed at every observed value of the confounders. For continuous confounders, a similar condition can be expressed using densities.

3. **Identification**: Under the assumptions above, we can re-write our casual estimand of interest (1) in terms of aspects of the joint distribution of the observed data. It is useful to follow these mathematical steps, to appreciate why we need the assumptions above. First we re-write our estimand using iterated expectations as:

$$E\{Y - Y(0)\} = E(Y) - E[E\{Y(0)| \mathbf{C}\}].$$

Then, under the conditional exchangeability assumption, $Y(0)$ is independent of X given $\mathbf{C}$, which gives us the licence to insert $X = 0$ on the right-hand side of the conditioning line in the second term, which means that our estimand is re-written as:

$$E(Y) - E[E\{Y(0)| X = 0, \mathbf{C}\}].$$

Then, under the consistency assumption, we can replace $Y(0)$ by Y on the left-hand side of the conditioning line in the same term, giving us:[6]

$$E\{Y - Y(0)\} = E(Y) - E\{E(Y| X = 0, \mathbf{C})\}.$$

[6] Note that consistency, as we stated it, is stronger than what is required to achieve this step. It would be sufficient to have consistency in conditional expectation given $\mathbf{C}$, *i.e.* that $E\{Y(0)| X = 0, \mathbf{C}\} = E(Y| X = 0, \mathbf{C})$. This relaxation would be important for the mixture intervention discussed for $Y(1)$ above, since consistency would be violated for such an intervention, but the weaker $E\{Y(1)| X = 1, \mathbf{C}\} = E(Y| X = 1, \mathbf{C})$ would be satisfies provided the interventions contributing to the mixture were all non-invasive.

This is important, since—using our first two assumptions—we have been able to write our estimand of interest, which involves data that we *don't* have, *i.e.* data from a hypothetical world in which there are no UTIs for pregnant women, in terms of aspects of the distribution of the data that we *do* have.

If all confounders were discrete, our estimand (1) could be estimated from a sufficiently large dataset on X, Y and $\mathbf{C}$ as long as the positivity assumption also holds. Positivity would ensure that $E(Y|X = 0, \mathbf{C} = \mathbf{c})$ could be non-parametrically estimated, for every observed value $\mathbf{c}$ of the confounders, as the observed prevalence of LBW among infants with unexposed mothers with level $\mathbf{c}$ of the confounders. In practice, parametric (or semiparametric) estimation is more typically done, although this can be made very flexible using machine learning [33].

4. **Statistical estimation and inference**: There are many estimation options, some traditional and some novel proposed by the FACE—this is where propensity scores, inverse probability weighting, doubly-robust methods, and doubly-robust methods that incorporate machine learning algorithms come in—but the simplest option is to estimate $E(Y|X = 0, \mathbf{C})$ directly from a parametric regression model fitted to the observed data. In this example, we could regress Y on $\mathbf{C}$ among the uninfected mothers, *e.g.* using a logistic regression model (including interactions/non-linearities as the data suggest, being liberal in as much as the sample size allows, since prediction of the potential outcome is the goal here, rather than interpretability of the model coefficients). We would then use the estimated coefficients from this model to predict the probability of $Y = 1$, under hypothetically no infection, based on $\mathbf{C}$, for each individual in the dataset (uninfected and infected). The average of these predictions would be our estimate of $E\{E(Y|X = 0, \mathbf{C})\}$. Subtracting this from the empirical mean of Y would give us our estimate of our causal estimand of interest, the prevalence of LBW attributable to UTIs in this population. We would also obtain the SE, CI *etc.* using either analytic or empirical (*e.g.* bootstrap) methods.

5. **Interpretation, misinterpretation and sensitivity analysis**: The effect estimates (and associated measures of statistical uncertainty) are interpreted, but, in particular, plausible violations of the assumptions are discussed explicitly in order to investigate possible misinterpretations. More formally, the sensitivity of the inference to possible violations of the assumptions can be assessed.

The FACE is of course concerned with a far larger set of problems than is represented by this very simple example, but the principles above carry through to settings with repeated exposures, time-dependent confounding, instrumental variables, effect modification, interaction, mediation, time-to-event outcomes, semiparametric estimation based on propensity scores *etc.*

A comparison with the 'traditional' approach

Traditionally, epidemiologists would certainly devote attention to establishing what is the question of interest. They would not usually, however, formally convert this into an estimand of interest that can be expressed mathematically as we did in feature 1. above. In some settings, things may be obvious enough for skipping 1. not to matter. However, skipping 1. can lead to serious mistakes in the later listed features. For example, the concepts of effect modification and interaction may not be distinguished, estimands such as the effect of the exposure *in the exposed* may not be easy

to articulate, and differences between possible direct effects (*e.g.* controlled vs. natural) may be overlooked. More seriously, without a clear mathematical estimand, there is the risk of calling the result of certain calculations, *e.g.* product of coefficients in mediation analysis, two-stage least squares in IV estimation, an indirect effect or a causal effect, respectively, even when such an interpretation is not allowed. Since feature 1. would traditionally be skipped, feature 3. (a mathematical link, via assumptions, between the estimand and the data) could not be attempted, and therefore a formal statement of assumptions (feature 2.) is not usually made. Traditionally, the assumption in 2(b)—no unmeasured confounding—would be stated, albeit more vaguely, but the consistency and positivity assumptions are usually overlooked. Without a clear idea of the assumptions made, sensitivity analyses (feature 5.) cannot reliably be carried out.
